# Epidemiological and Spatiotemporal Descriptive Analysis of Patients with Nonsyndromic Cleft Lip and/or Palate: A 12-Year Retrospective Study in Southern Iran

**DOI:** 10.1155/2023/7624875

**Published:** 2023-04-19

**Authors:** Nima Farshidfar, Shabnam Ajami, Sarina Sahmeddini, Ali Goli, Hamid Reza Foroutan

**Affiliations:** ^1^Orthodontic Research Center, School of Dentistry, Shiraz University of Medical Sciences, Shiraz, Iran; ^2^Student Research Committee, Shiraz University of Medical Sciences, Shiraz, Iran; ^3^Department of Sociology & Social Planning, Shiraz University, Shiraz, Iran; ^4^Department of Surgery, Laparoscopy Research Center, Shiraz University of Medical Sciences, Shiraz, Iran

## Abstract

**Objectives:**

The current study is aimed at evaluating epidemiological characteristics and spatiotemporal distribution of cleft lip and/or palate (CL/P) in the south of Iran.

**Methods:**

Data were extracted from the 1840 medical records of patients who were referred to the Cleft Lip and Palate Center of Shiraz University of Medical Sciences, from January 1, 2011, to September 1, 2022. The collected variables included demographic data (gender, birth date and season, place, birth order, and weight), cleft types and the subtypes, parental information (health status, education level, marital status, and age during the pregnancy), and other basic parameters. The chi-square test at a significance level of 0.05 was used to analyze collected data. The geographic information system (GIS) analysis was also used for analyzing the spatial distribution of CL/P patients.

**Results:**

Based on our inclusion criteria, 1281 nonsyndromic patients were included in this study. The most common type was cleft lip and palate (CLP) with 48.32%, whereas cleft palate (CP) and cleft lip (CL) accounted for 40.75% and 10.93% of the patients, respectively. There was a progressive increase in the frequency of all types of clefts, and most of them were male (*P* ≤ 0.001). The urban population outnumbered the rural ones in all provinces. Parents were mostly healthy (>80%) with low educational status (47.5%). Most born CL/P patients were from consanguineous marriages (58.9%), especially between first-degree relatives. A majority of CL/P patients (73.1%) were born in the first two gestations with a birth weight of 2500-4000 g (77.4%). Most infants with CL/P (84.3%) were born from mothers who had at least one of the predisposing factors.

**Conclusion:**

In this study, the frequency of cleft types and subtypes was similar to the existing literature. However, high rate of consanguineous marriage, especially between first-degree relatives, was the most notable feature of this population.

## 1. Introduction

Cleft lip and/or palate (CL/P) is one of the most frequent congenital abnormalities occurring in the orofacial region [1, 2]. Patients with CL/P are usually associated with various complications including speech and hearing impairments, malocclusions, significant psychiatric issues, and social handicaps (e.g., impaired sucking and the ensuing inability to flourish) [3, 4]. In this context, speech and hearing impairments are of the most prominent complications in CL/P patients [3, 4]. The existence of different types of clefts naturally causes speech impairment in these patients [5]. Additionally, patients with CL/P are often born with otitis media with effusion (OME) which is brought on by the dysfunction of the Eustachian tube muscles. As a result, this tube cannot balance pressure and drain secretions, which leads to OME and conductive hearing loss [6, 7]. Even a minor degree of hearing impairment may have a profoundly deleterious effect on speech development as well [8]. Based on a recent meta-analysis of more than 50 studies, the global prevalence of CL/P was 0.30-0.45 in every 1000 live births [9]; however, the published data on the prevalence of CL/P from different parts of the world are extremely diverse [10]. In Iran, it was projected to be more common than in other countries (1.24 in every 1000 live births) [11].

This congenital abnormality is also divided into syndromic and nonsyndromic types. Nonsyndromic CL/P comprises ~70% of the cases and is considered to be a multifactorial disorder caused by the interaction of environmental and genetic factors [12, 13]. However, due to ethnic or regional variances, the precise cause of the nonsyndromic type is still not entirely understood [14]. Since their treatment involves intricate and multidisciplinary approaches, preventive measures through the identification of environmental risk factors are of the utmost importance [15]. Several environmental risk factors (e.g., active/passive maternal smoking) have been identified as consistent ones in various studies [16]. Nevertheless, other environmental risk factors are attributed to a geographically distinct population [17]. Additionally, gene-environment interactions may also differ due to genetic variants in different populations [12]. Therefore, population-specific studies are essential to take into account the variations in environmental susceptibility in the different regions [12].

Considering the high prevalence of CL/P and wide ethnic or regional differences in Iran [11], it is crucial to evaluate the distribution of CL/P and its associated risk factors in different regions of Iran. Previous studies in Iran have analyzed small datasets in local locations [18, 19] or larger datasets in different locations [20, 21]. However, none of them provided a comprehensive overview of the nonsyndromic CL/P in southern Iran due to a lack of information. Rajabian and Aghaei reviewed only 119 records of nonsyndromic CL/P from 1993 to 2003 in southwest Iran and found no significant correlation between gender and the laterality pattern of this deformity with cleft type [18]. More recently, Galeh et al. also conducted a retrospective study on 1500 CL/P patients born between 2010 and 2020 in northwest Iran and evaluated only their demographic characteristics and distribution of accompanying anomalies [21].

Furthermore, the distribution of CL/P can also be evaluated through geographical epidemiology using geographic information system (GIS). GIS is a practical method that provides an opportunity for generating assumptions and identifying the effects of different factors such as environmental, social, cultural-behavioral, and genetic factors on the spatial pattern of diseases [22, 23]. Although there is no study regarding the application of GIS for demonstrating the spatial distribution of CL/P patients in Iran yet, this method has been employed in other countries [17, 24]. Pradubwong et al. in 2010 used a GIS approach to study the distribution and statistics of patients with CL/P in the northeast region of Thailand [24]. More recently, Zhu et al. in 2020 conducted a descriptive analysis to illustrate the spatiotemporal distribution of the prevalence of CL/P born between 2015 and 2018 in Guangdong province, China [17].

Overall, the present study is aimed at contributing to the descriptive analysis of numerous epidemiological variables and environmental factors associated with nonsyndromic CL/P patients over the past 12 years in southern Iran. We also employed GIS for the first time to collect and analyze the spatiotemporal distribution of this abnormality in this region. This information would provide references for proper resources to construct primary preventive programs and direct counseling, based on the specific data for CL/P anomaly extracted from every part of this region.

## 2. Materials and Methods

### 2.1. Study Design, Settings, and Data Source

This retrospective cross-sectional descriptive study was conducted according to the STROBE guideline [25]. Data were collected from the database of the Cleft Lip and Palate Center, Orthodontic Research Center, Shiraz University of Medical Sciences, Shiraz, Iran. This center was established on January 1, 2011, as the only, first, and largest Cleft Lip and Palate Center in southern Iran, to give service to infants, children, and adults who are suffering from this congenital abnormality and are gravely in need of urgent and holistic care.

### 2.2. Study Size and Participants

Since we used the census, there was no sample size calculation for this study. Data were extracted from the 1840 medical records of patients who were referred to the Cleft Lip and Palate Center, Orthodontic Research Center, Shiraz University of Medical Sciences, from January 1, 2011, to September 1, 2022. These medical records were previously documented by orthodontic specialists working as team members when the patients were examined for the first time in our center. In this study, we only extracted the relevant data accurately from the database of the registered CL/P patients.

Patients with diagnosed nonsyndromic CL/P who were born in southern provinces of Iran (Fars, Kohgiluyeh and Boyer-Ahmad, Boushehr, Hormozgan, and Khoozestan) were the population of this study. Otherwise, patients were excluded from the study if they have one of the following criteria: (1) patients with syndromic CL/P which is accompanied by deformities of other tissues and organs in the body, (2) patients whose records were incomplete or missing, (3) non-Iranian patients, (4) patients who were born in other areas rather than the included provinces, and (5) patients who were registered for any abnormalities other than CL/P.

### 2.3. Variables

A checklist specifically designed based on the variables related to the purposes of this study was used to extract the data (Table 1). This checklist gathered specific information from each patient's medical record who had met the eligibility criteria to be included in this study.

### 2.4. Ethical Consideration

The study protocol complied with the Declaration of Helsinki and was approved by the Ethics Committee of Shiraz University of Medical Sciences (IR.SUMS.REC.1400.872).

### 2.5. Geographical Analysis

ArcGIS software (version 10.8.2) was used for depicting and analyzing the spatial distribution of CL/P patients to offer much more realistic patterns alongside statistical analysis. Supplementary Figure 1 shows the location of the southern provinces of Iran and the regional distribution of their counties. In this regard, a choropleth map was provided to show the frequency of CL/P patients based on the counties where they were born. The other maps were also designed to illustrate the cleft types, gender, birth date, birthplace, and marital relationship of the parents based on the province where they were born. Furthermore, each province was delineated separately, and then, exploratory spatial data analysis (ESDA) on the scattering pattern of CL/P patients was done. In this regard, hot-spot and cold-spot analyses were performed to delineate the spatial cluster of CL/P patients based on the Getis-Ord Gi statistic using a fixed distance band in ArcGIS software (version 10.8.2) [26].

### 2.6. Statistical Analysis

The results were presented as absolute numbers (*N*) and as percentages (%). The chi-square test at a significance level of 0.05 was performed using IBM® SPSS® Statistics 26.0 for the data analysis.

## 3. Results

**Figure 1:**
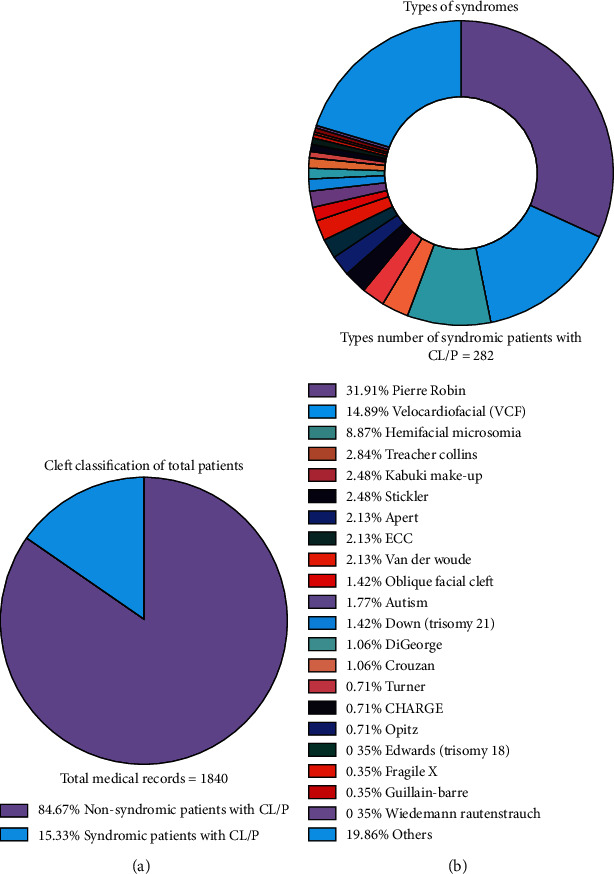
(a) Frequency of patients with CL/P based on the presence of syndrome and (b) type of syndrome.

**Figure 2:**
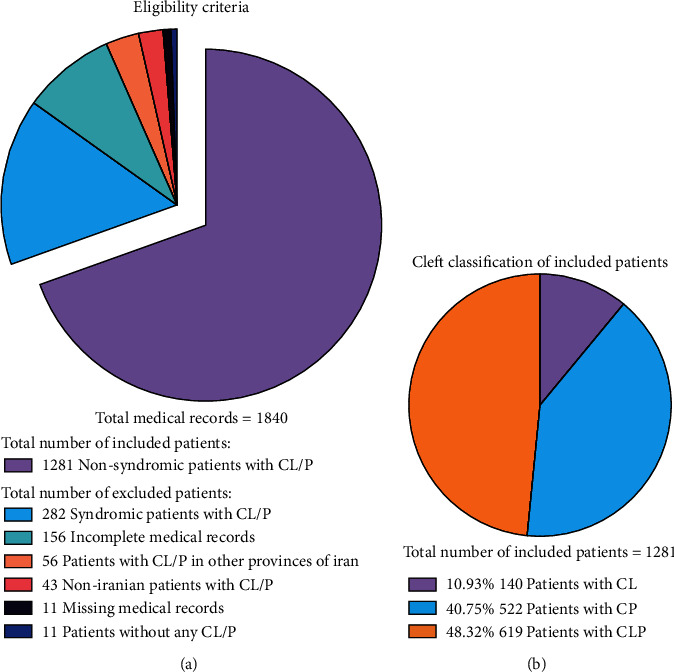
(a) Total number of CL/P patients based on the inclusion/exclusion criteria. (b) Frequency and the total number of included CL/P patients according to their cleft type.

**Figure 3:**
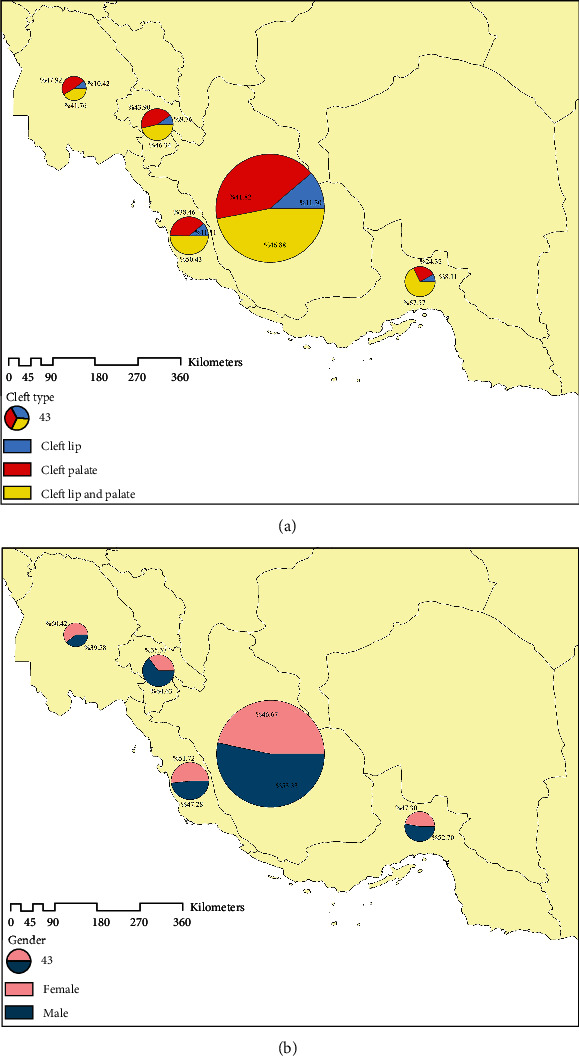
Spatial distribution of CL/P patients in terms of their (a) cleft type and (b) gender.

Data were obtained from the medical records of 1840 patients with CL/P. Regardless of the eligibility criteria, 1558 (84.67%) patients were nonsyndromic, and 282 (15.33%) of them were syndromic (Figure 1(a)). Among syndromic patients, Pierre Robin, velocardiofacial (VCF), and hemifacial microsomia with 31.9%, 14.89%, and 8.87%, respectively, were the most common types of syndromes associated with CL/P. Other types of syndromes observed among syndromic patients were listed according to their frequencies in Figure 1(b). However, based on our inclusion criteria, 1281 nonsyndromic patients were included in this study, and the others were excluded due to the mentioned reasons in Figure 2(a). Out of these 1281 nonsyndromic patients with CL/P, the most common type was complete cleft lip and palate (CLP) with 48.32% (*n* = 619), whereas isolated cleft palate (CP) and isolated cleft lip (CL) accounted for 40.75% (*n* = 522) and 10.93% (*n* = 140) of the patients, respectively (Figure 2(b)). The spatial distribution of these patients according to their cleft type also showed the same distribution for southern provinces, except for Hormozgan with exceeding cases of CLP (67.57%) and Khoozestan with higher cases of CP (47.92%) (Figure 3(a)).

### 3.1. Cleft Types and Subtypes

Table 2 depicts the frequencies (*N*, %) of each subtype of CL, CP, and CLP. Among patients with CL, incomplete CL (79.3%) was more prevalent than complete ones (20.7%), and unilateral CL (87.8%) outnumbered bilateral one (12.2%). Overall, the majority of patients with CL were left-sided (47.8%). Among patients with CP, the most common cleft pattern was the incomplete one (79.5%), and submucosal and complete types comprised 19.33% and 1.2% of the patients with CP, respectively. Similar to the patients with CL, most CLP cases were unilateral (69.8%), and they more commonly occurred on the left side (43.8%).

### 3.2. Gender

The distribution of gender for nonsyndromic patients with CL/P is summarized in Tables 3 and 4. Overall, 53.1% of the patients were males, while 46.9% of them were females. The spatial distribution of these patients in terms of their gender also shows the same pattern in southern provinces, except for Boushehr (51.72%) and Khoozestan (60.42%) with more female cases (Figure 3(b)). There was also a significant difference between patients with CL, CP, and CLP regarding their gender (*P* ≤ 0.001). CL and CLP tend to occur more in males than females (55.7% : 44.3% and 61.4% : 38.6%, respectively). On the contrary, CP tends to occur more in females (57.5%) than males (42.5%) (Table 4).  Table 3 (A, B, C, and D) also elaborately illustrates the frequencies (*N*, %) of each subtype of CL, CP, and CLP according to gender.

### 3.3. Birth Dates and Birth Season

**Figure 4:**
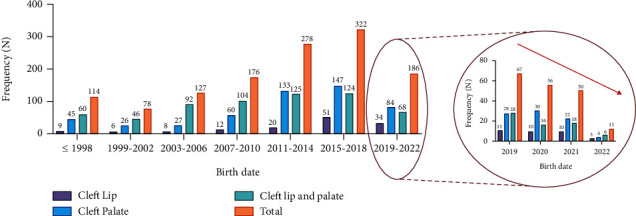
Temporal distribution of patients with CL, CP, and CPL according to their birth date.

The temporal distribution of nonsyndromic patients with CL/P according to their birth dates and birth seasons is summarized in Table 4. We divided birth dates into the following categories with a 4-year period: ≤1998, 1999-2002, 2003-2006, 2007-2010, 2011-2014, 2015-2018, and 2019-2022. It was shown that there was a significant difference between patients with CL, CP, and CLP regarding their birth dates (*P* ≤ 0.001).  Figure 4 visually demonstrates a progressive increase in the frequency of all cleft types until 2018 and a downward trend in the frequency of referring patients with CL/P as of 2019. Until the end of 2010, CLP had a much higher predominance over the other two types; however, the frequency of patients with CP outnumbered those with CLP from the beginning of 2011. Table 4 also clearly shows that the frequency of all the cleft types is roughly 25% in all birth seasons.

### 3.4. Birthplace

**Figure 5:**
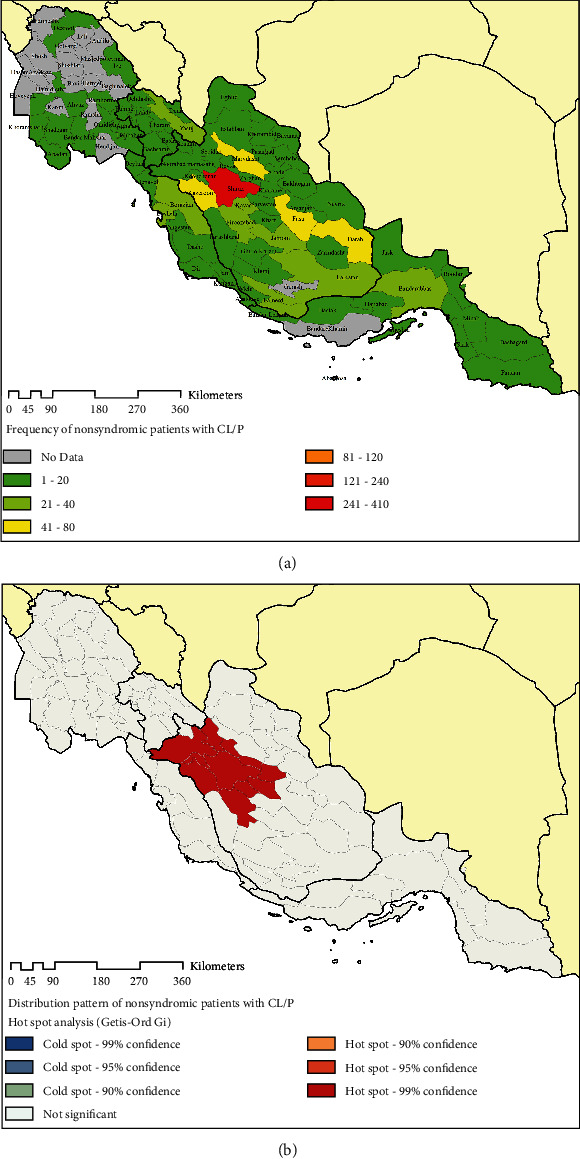
(a) Spatial distributions of the frequency and (b) distribution pattern of nonsyndromic patients with CL/P in southern Iran.

**Figure 6:**
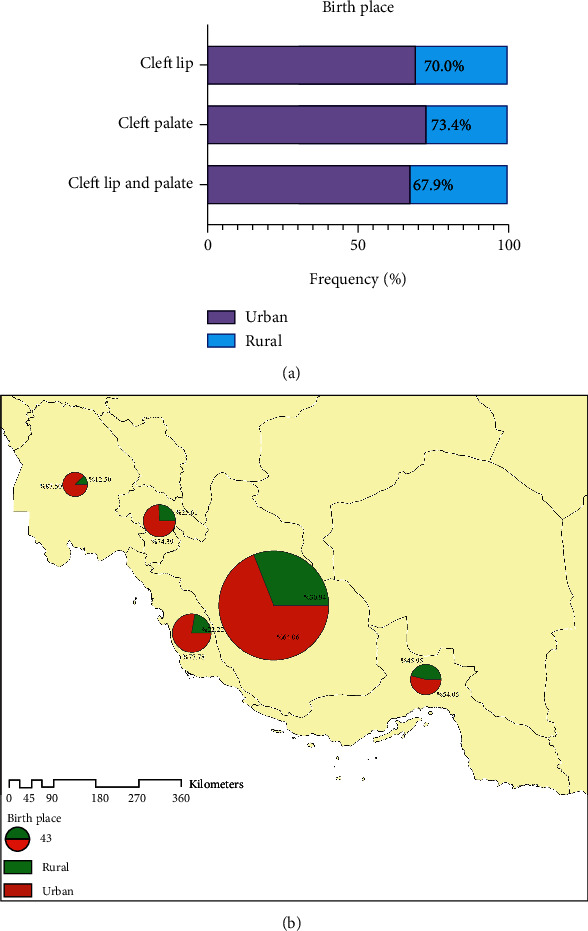
(a) Frequency of patients with CL, CP, and CLP and (b) spatial distribution of CL/P patients in terms of their birthplace.

Figure 5(a) illustrates the spatial distribution of nonsyndromic patients with CL/P who attended our center for cleft team management, according to their provinces and counties where they were born in.  Table 5 also depicts that Fars province had the highest frequency (75%), while Khoozestan province had the lowest (3.7%). Most spatial heterogeneity of the frequency of CL/P across the study period was observed in Fars. Being the capital city of Fars, Shiraz County, where our Cleft Lip and Palate Center is located, had the highest frequency rates of referred CL/P (Figure 5(a)). According to the hot-spot and cold-spot analyses, Figure 5(b) clearly shows spatial patterns of high concentration of CL/P patients in Shiraz and its neighboring counties, which was statistically significant (*P* ≤ 0.01) in comparison to other counties. Additionally, Figure 6(a) depicts whether these patients come from an urban or rural area, and Figure 6(b) also indicates that the urban population outnumbers the rural ones in all provinces. The overall frequency of CL, CP, and CLP in urban areas was higher than that in rural areas.

### 3.5. Maternal and Paternal Age, Health Status, and Educational Level

The distribution of maternal and paternal age, health status, and educational level for nonsyndromic patients with CL/P is summarized in Table 6. There was a significant difference between patients with CL, CP, and CLP regarding their maternal and paternal age as well as the maternal educational level (*P* ≤ 0.05). Maternal age ranges from 25 to 29 were the most common age range for patients with CL (34.3%), CP (35.1%), and CLP (33.3%), and no specific pattern was observed regarding maternal age. On the other hand, the paternal age of more than 35 was the most common age range for patients with CP (39.8%) and CLP (33.9%); however, the age ranges from 30 to 34 were the most common one for patients with CL (43.6%). Overall, with increasing paternal age, CL/P tended to occur more frequently. Regarding the maternal and paternal health status, it was found that more than 80% of mothers and more than 90% of the fathers of patients with CL, CP, and CLP were healthy and had no systemic or underlying disease. We also found that infants born by mothers and fathers with an educational level of middle school or less accounted for a relatively large number of CL/P patients (47.5% and 45.8%). Overall, CL/P tended to occur more frequently with decreasing maternal and paternal educational levels.

### 3.6. Marital Status of the Parents

**Figure 7:**
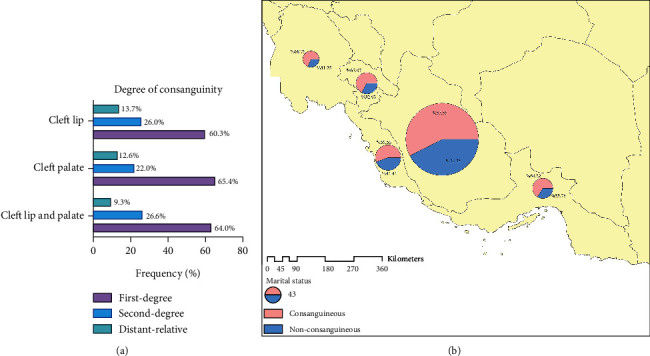
(a) Frequency of degree of consanguinity among CL/P patients born from consanguineous marriages. (b) Spatial distribution of CL/P patients in terms of their marital status.

The distribution of marital status of the parents for nonsyndromic CL/P is summarized in Table 6. It was observed that the percentages of born cases with CL/P from consanguineous marriages (58.9%) were greater than nonconsanguineous ones (41.1%). The spatial distribution of these patients according to their marriage type also shows the same pattern in all of the southern provinces, especially in Khoozestan (68.75%), Kohgiluyeh and Boyer-Ahmad (67.07%), and Hormozgan (66.22%) in which the rate of consanguineous marriages was higher (Figure 7(b)). In this regard, consanguineous marriage between first-degree relatives had the highest frequency among parents of CP (65.4%), CLP (64%), and CL (60.3%) patients (Figure 7(a)). The lower the degree of consanguinity becomes, the less frequent all types of clefts become (Figure 7(a)).

### 3.7. Time of Diagnosis and Type of Delivery

The distribution of time of diagnosis and type of delivery for nonsyndromic patients with CL/P is summarized in Table 7. There was a significant difference between patients with CL, CP, and CLP regarding their time of diagnosis and type of delivery (*P* ≤ 0.05). It was found that 85% of CL/P patients were born to mothers who had a history of prenatal ultrasound utilization; however, only 12.7% of CL/P patients were diagnosed during pregnancy, and a majority of them (87.3%) were diagnosed after the delivery. Regarding the delivery method, there is not much difference between vaginal birth (48.9%) and cesarean section (51.1%) for giving birth to newborns with CL/P.

### 3.8. Birth Order and Birth Weight

The distribution of birth order and birth weight for nonsyndromic patients with CL/P is summarized in Table 7. We observed that a majority of CL/P patients (73.1%) were born in the first two gestations. Additionally, the frequency of patients with CL/P decreases as the birth order increases. Contrary to the birth order, there was a significant difference between patients with CL, CP, and CLP regarding their birth weight (*P* ≤ 0.05). Infants with a birth weight of 2500-4000 g accounted for the majority of CL/P patients (77.4%).

### 3.9. Predisposing Factors and Familial History

**Figure 8:**
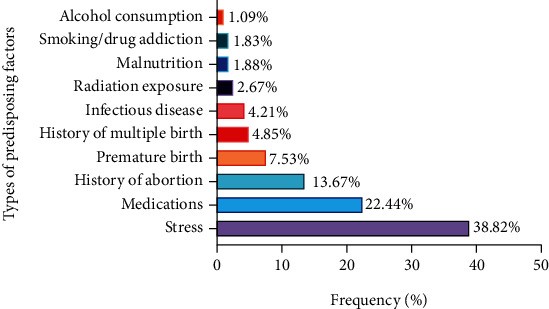
Frequency of different types of predisposing factors among mothers of CL/P patients during the first trimester of their pregnancy.

The distribution of predisposing factors (during the first trimester of pregnancy), familial history of CL/P, and other congenital abnormalities for nonsyndromic patients with CL/P is summarized in Table 8. It was shown that there was a significant difference between patients with CL, CP, and CLP regarding their maternal predisposing factors and familial history of CL/P (*P* ≤ 0.05). Most infants with CL/P (84.3%) were born to mothers who were exposed to at least one predisposing factor during the first trimester of their pregnancy.  Figure 8 shows predisposing factors observed among them according to their frequency. Stress (38.82%), taking medications (22.44%), and a history of abortion (13.67%) were the most common predisposing factors associated with CL/P. Besides, mothers who were passively exposed to smoking accounted for 33.3% of patients born with CL/P. It was also demonstrated that the familial history of CL/P accounted for 26.7% of patients with CL/P; however, a rare number of CL/P patients (2.8%) had a familial history of other congenital abnormalities.

## 4. Discussion

CL/Ps are considered as a nonsyndromic congenital anomaly, but they can be accompanied by syndromes and be one of their manifestations [27]. Our study showed higher cases of nonsyndromic CL/P patients (84.67%) in comparison to syndromic ones (15.33%), which was similarly reported in the literature [28, 29]. The literature commonly stated that nonsyndromic and syndromic forms comprise approximately 70% and 30% of CL/P patients, respectively [28, 29]. Nevertheless, various authors have reported that the incidence of associated anomalies could vary from as low as 4.3% to as high as 63.4% [30, 31]. Additionally, the most frequent syndromes observed in our population, with decreasing order, were Pierre Robin (31.9%), VCF (14.89%), and hemifacial microsomia (8.87%). Similarly, Pierre Robin syndrome was the most frequent syndrome in Pakistan [32], Saudi Arabia [33], and Hong Kong [34] populations. Also, in a northwest Iranian population, Pierre Robin and VCF were the first and second most frequent syndromes observed [21]. However, these findings differ from the previous ones reported in 2014 in which Van der Woude was declared to be the most common syndrome associated with CL/P, accounting for ~2% of all CL/P cases [35], and are in dark contrast with another study in which other syndromes such as the Edwards syndrome (28%) and the brain-lung-thyroid syndrome (17%) were the most common ones [1]. These disparities could be related to the methodology used to diagnose the syndromes, as well as variances in the base populations and ethnicities [36]. Overall, there are no accurate data available because the clinical diagnosis of these CL/P-associated syndromes has a large and variable phenotypic spectrum. Clinicians must be trained, and molecular studies are needed to confirm the diagnosis and determine the specific prevalence of each disease in each patient in the Iranian population [36].

In our study, the distribution of the three types of this congenital abnormality was 10.93% for CL, 40.75% for CP, and 48.32% for CLP. The spatial distribution of these patients also showed the same pattern in all provinces, except for Hormozgan and Khoozestan with exceeding cases of CLP (67.57%) and CP (47.92%), respectively. These results are in contrast with a study in Pakistan, which reported the distribution of 42%, 24%, and 34%, for CL, CP, and CLP, respectively [32]. However, the predominance of CP in our study was similar to the study conducted in northwest Iran (41%) [21], and the frequency of CLP in our study was also similar to other studies in southwestern (45.4%) [19] and northeast (50%) [20] of Iran. Additionally, our results are consistent with the studies in Syria [14] and Japan [37]. It seems that environmental factors play a critical role in the differences between the various studies [38] and different regions in our study. In terms of laterality, patients with unilateral CL (87.8%) and unilateral CLP (69.8%) outnumbered the bilateral ones in our study. Additionally, the majority of our CL (47.8%) and CLP (43.8%) cases had left unilateral cleft. These findings conform with several research results showing that unilateral cleft is more frequent than bilateral one and commonly occurs on the left side [14]. One rationale for the higher occurrence on the left side is that facial artery development is delayed on the left side compared to the right one [39]. Nonetheless, this has not been fully validated [39]. It was also shown that the majority of CP cases (79.5%) in our population were incomplete which is completely consistent with the results of another study in Estonia [40].

Male and female infants were 53.1% and 46.9% of all CL/P subjects in this study, respectively. The spatial distribution of these patients also shows the same pattern for each southern province, except for Boushehr (51.72%) and Khoozestan (60.42%) with more female cases. These variations may be justified by the environmental factors that are probably involved in geographically distinct populations [17]. Additionally, our results showed a slight predominance of males in CL (55.7%), while CLP had a much higher predominance in males (61.4%) compared to females. On the other hand, female infants accounted for almost most cases in CP (57.5%) patients. Other investigations reported a larger incidence of CP in females [41–43], as well as a higher incidence of CLP in males [12, 42, 43], with one study finding a male dominance for CL [12]. Contrary to our study, one paper showed that CL was much higher among females (70%), while another found no significant differences in the incidence of CL between the genders [44].

Temporal distribution in our study illustrated a progressive increase in the frequency of all types of clefts up to 2018. According to the GBD 2020 study regarding the global burden of oral disorders, it was shown that the global count of cases with CL/P has increased from 1990 to 2019 [45]. However, as of December 2019 due to the COVID-19 pandemic [46, 47], the number of patients with CL/P referring to our Cleft Lip and Palate Center decreased. Additionally, as of 2011, it seems that the trend regarding the frequency of cleft types is changing, and CP is becoming more frequent than the other cleft types. A recent investigation conducted on 1500 CL/P patients born between 2010 and 2020 in the northwest of Iran also showed that CP patients comprise the majority of cases (41%) [21]. Our results also demonstrated no relation between seasonal birth and nonsyndromic CL/P types, which is confirmed by other investigations in the Iranian population [20, 48]. However, a larger proportion of nonsyndromic CL/P was seen during the spring and summer in Puerto Rico [49] and through the spring in the Chinese population [17]. These variations in results may be attributed to environmental factors that are probably involved in geographically distinct populations [17].

For spatial distributions of the frequency, we observed heterogeneity of CL/P across the counties in our study. Previous studies also indicated that the global distribution of the CL/P is unbalanced and there is spatial heterogeneity in this regard [10]. Uneven distribution of CL/P in a single country is also possible due to various environmental factors in different regions [17]. The spatial analysis also showed that Shiraz and its neighboring counties created a cluster that had the most frequent cases of CL/P. The possible justification is that Shiraz is the most highly populated county in the region [50]. It seems that establishing a Cleft Lip and Palate Center in Shiraz was a must in providing diagnostic and treatment services to CL/P patients from nearby provinces. On the other hand, long distances and the high cost of traveling [51] may also justify why those who were born outside the Fars province are less likely to refer to our center. The existence of another Cleft Lip and Palate Center in Esfahan explains this form of distribution from Shiraz to some distant counties [3], especially related to the Khoozestan province, with the relatively low number of CL/P patients and the lack of proper data in our study. Additionally, the frequency of CL/P in our study was higher in urban areas rather than in rural ones. Corroborating our results, an investigation during 2005-2014 in China also showed that the prevalence of CL/P in urban areas was much higher than that in rural ones [52]. The possible reason could be that people in urban areas can more easily afford healthcare services, and consequently, a higher number of CL/P cases come from these areas [53]. Also, another reason could be maternal exposure to ample amounts of ambient air pollutants in urban areas than that in rural ones [54]. Nevertheless, Messer et al. demonstrated that living in rural areas was associated with an increased adjusted risk of CL/P and could increase its occurrence [55].

By analyzing the parental characteristics of infants with CL/P in this present study, there was no association between advanced maternal age and the occurrence of CL/P. In line with our study, some others conducted in Canada, Iran, the Netherlands, and South America also did not find an association between advanced maternal age and CL/P [56, 57]. On the other hand, we found an association between advanced paternal age and the occurrence of CL/P in our study. In a recent study among nonsyndromic CL/P patients in Central Africa, paternal age above 35 years was identified as a risk factor for CL/P [12]. However, in the multivariate analysis, it was not retained as a significant risk factor. Bille et al. also demonstrated that high paternal age was associated with CL/P; however, the maternal age did not have the same effect [58]. Nevertheless, older ages may be associated with cumulative changes in gametes over time (lifelong medication usage, incidence of chronic diseases, and socioeconomic conditions), as well as reduced uterine selectivity for faulty embryos and increased placental permeability to teratogenic substances [12, 59].

We also discovered that just 18.3% of mothers with CL, CP, or CLP had a systemic disease. Mirilas et al. found that maternal systemic disease did not significantly enhance the incidence of CL/P in infants [60]. Taghavi et al. and Krapels et al., on the other hand, found that maternal systemic disease enhanced the likelihood of CL/P in offspring [61, 62]. Our research also indicated that low parental educational levels appeared to be more frequent in children with nonsyndromic CL/P. This is consistent with the findings of Krapels et al. and J. Dvivedi and S. Dvivedi, who found that people with lower levels of education smoked more and ate less healthily than people with higher educational degrees [62, 63]. Lin et al. also considered low parental educational levels as a risk factor for having a child with nonsyndromic CL/P [64].

The results from our study showed that most of the CL/P patients come from consanguineous marriages (58.9%), and marriage between first-degree relatives had the highest frequency among them. Consanguineous marriage is a major feature of family systems in Iran [65]. Saadat et al. showed that the overall rate of consanguineous marriages was 38.6% among a total sample of 306 343 Iranian couples. It was also shown that the frequency of this type of marriage was even higher in the southern parts of Iran (43.8%) [65]. Corroborating our findings, Silva et al. in Brazil and Sabbagh et al. in Saudi Arabia found that parental consanguinity was associated with the occurrence of nonsyndromic CL/P and also showed that this occurred more frequently among first-degree relatives [66, 67]. This association could be attributed to a recessive genetic component [68]. Additionally, since the rate of consanguineous marriages in all southern provinces is relatively high, the importance of genetic counseling before this type of marriage should be taken into consideration [68].

Our study demonstrated that a majority of mothers had a history of prenatal ultrasound utilization (85%). However, only 12.7% of them were diagnosed with having an infant with CL/P during their pregnancy period. In this regard, a lack of proper knowledge regarding craniofacial embryology and consequently utilization of ultrasound at an inappropriate period of gestation can lead to misdiagnosis of CL/P [69]. Furthermore, isolated CP is rarely identified prenatally because the palate has a dome-shaped structure and is encircled by osseous tissues, making visualization difficult [70]. Besides, utilizing conventional 2D ultrasounds and lack of access to 3D ultrasounds could certainly lead to a decreased frequency of CL/P being diagnosed antenatally [70]. Additionally, it seems that since the majority of the mothers were not diagnosed in terms of having a child with CL/P before their gestations, there was no preferred delivery method (vaginal or cesarean section) reported in our study.

Generally, there is no consensus on whether birth order has any association with CL/P [71]. Our study showed no relation between higher birth order and CL/P, and a majority of CL/P patients were born in the first two gestations (73.1%). In line with our study, Martelli et al. found no significant statistical association between these variables in the Brazilian population as well. Additionally, among their CL/P patients, 74% were born within the first two gestations [72]. Conforming to our results, investigations carried out in Iran [19], France [73], and Sri Lanka [74] also did not report any associations between birth order and CL/P. However, Vieira and Orioli conducted a meta-analysis regarding the association of birth order and CL/P and discovered a statistically positive link between these conditions [71]. Furthermore, most of the assessed patients in our study had a normal birth weight (77.4%), which is also conformed with another study in which no evidence of low/high birth weight was found in infants with CL/P [40]. On the contrary, some studies demonstrated that children with CL/P presented smaller body dimensions when compared to controls [75, 76].

Our study also showed that 84.3% of infants with CL/P were born to mothers who were exposed to at least one predisposing factor during the first trimester of their pregnancy. Stress was the most frequent predisposing factor associated with CL/P. Wallace et al. found that mental or emotional stress regards to be a likely risk factor in the occurrence of CL/P [77]. It was also mentioned that continuously high stress levels may lead to abnormal fetal development and consequently could potentially cause changes in the physiology of the developing child [77]. The second predisposing factor was taking medications. Previous literature associated the use of drugs (e.g., antibiotics, corticosteroids, antidepressant, anticonvulsant, and barbiturates) during pregnancy with CL/P occurrence [3]. However, we believe that reporting an absolute result for all types of drugs is not statistically accurate, and it is recommended that each drug category be evaluated independently. The third most frequent predisposing factor was having a previous history of abortion, which was in line with the study of Cheshmi et al. revealing a potential correlation between this variable and the birth of a child with CL/P in subsequent pregnancies [3]. Shehan et al. recently evaluated the associations between prematurity and the development of CL/P and found that preterm newborns seem to be more susceptible to CL/P than full-term infants [78]. Zhu et al. also found that the frequency of CL/P is greater in mothers with multiple births than in those with single birth [17].

Other predisposing factors such as maternal diseases during the first trimester have been recently identified to play a significant role in the development of isolated CP [79]. Additionally, since organogenesis happens in the first trimester, exposure to radiation during this trimester would increase the chance of developing congenital abnormalities such as CL/P rather than in the second or third trimesters [80]. The remaining factors (i.e., malnutrition, smoking/drug addiction, and alcohol consumption) constitute less than 5% of the total predisposing factors associated with CL/P in our study. In this regard, malnutrition and smoking are well-documented problems associated with CL/P [16, 81, 82]. However, since smoking is not culturally accepted by Iranian women, its frequency as a predisposing factor is relatively low in this study. Nevertheless, a recently published meta-analysis indicates that maternal active smoking may play a moderate impact in the genesis of CL/P with an odds ratio of 1.42 (95% CI: 1.27, 1.59), and there was also limited evidence that smoking had a dose-response effect [82]. Also, another meta-analysis showed that maternal passive smoking exposure results in a 1.5-fold increase in the risk of nonsyndromic CL/P [16], similar to the magnitude of risk reported for maternal active smoking [82]. A recent meta-analysis also indicated that there was no statistically significant difference in the chance of having a child with a nonsyndromic CL/P between alcoholic and nonalcoholic women [83]. However, four of the studies included in this meta-analysis revealed a significantly elevated incidence of CL/P among mothers who consumed the most alcohol [83].

Our study also showed that the familial history of CL/P accounted for 26.7% of patients with CL/P. In this regard, it was recently stated that the children of pregnant women who have a positive family history of CL/P are at risk of recurrence [84]. Besides, it is indicated that there is a greater chance that the same type of cleft to be inherited by the offspring [85].

The study had also some limitations. First, there was no background population or controls to whom this population of patients can be compared because the data in this study was collected as consecutive cases coming from a nonprofit organization dedicated entirely to patients with any form of CL/P. Therefore, case-control studies are required to provide more precise evidence regarding the correlation between various epidemiological risk factors and CL/P disease. Second, this retrospective study provided only preliminary results regarding the frequency of various types of predisposing factors during the first trimester of pregnancy. In order to benefit from these results, future studies should focus on investigating these predisposing factors in depth by evaluating the exact types of infectious diseases or medications, assessing the exact time of the radiation exposure, providing further data regarding the history of abortion, etc. Third, this study was also a spatiotemporal descriptive analysis that merely explores the distribution of CL/P in the region and does not provide a clear relationship between the spatiotemporal parameters and the disease. Thus, a thorough analysis based on a multivariate approach should be carried out to identify the subgroups with a higher risk of CL/P in future studies. Fourth, data gathering through interviews after the birth of the patients seems to be more likely to result in lower-quality data, wrong replies because of forgetfulness, or unrealistic responses as a consequence of being overtaken by shyness. Furthermore, many patients with CL/P did not refer to our Cleft Lip and Palate Center as of December 2019 due to the COVID-19 pandemic, which could affect the results of our study.

## 5. Conclusion

The findings of this study could help to understand the epidemiological and spatiotemporal characteristics of CL/P and provide reliable descriptive material for subsequent research on this disease. Overall, within the limitations of this study, the following important conclusions can be drawn:

- CLP was the most frequent type of cleft, and the majority of CL and CLP patients were unilateral and left-sided
- CL and CLP tended to occur more in males, but CP occurred more in females
- Temporal descriptive analysis showed a progressive increase in the frequency of CL/P patients according to their birth dates; however, no association was observed between their birth seasons
- Spatial descriptive analysis also showed some variations in different provinces which may be attributed to environmental factors that are probably involved in geographically distinct populations
- CL/P in urban areas was higher than that in rural areas
- Parents were often healthy individuals with low educational levels
- Parents often had consanguineous marriages, and marriages between first-degree relatives had the highest frequency them
- Majority of CL/P patients were born within the first two gestations with a weight of 2500-4000 g
- Most infants with CL/P were born to mothers who were exposed to at least one predisposing factor during the first trimester of their pregnancy, and some of them had a positive familial history of CL/P

## Figures and Tables

**Table 1:** The checklist comprised variables regarding patients with CL/P.

Checklist		
Number	Variables	Types of variables
1	Type of the cleft	Cleft lip, cleft palate, or cleft lip and palate
2	Subtype of the cleft	Complete cleft, incomplete cleft, or submucosal cleft
3	Side of the cleft	Unilateral or bilateral
4	Location of the cleft	Left, right, or central
5	Gender	Male or female
6	Birth date	≤1998, 1999-2002, 2003-2006, 2007-2010, 2011-2014, 2015-2018, and 2019-2022
7	Birth season	Spring, summer, autumn, or winter
8	Birthplace	Province, county, and urban, or rural
9	Maternal and paternal age during pregnancy	24, 25-29, 30-34, or ≥35
10	Maternal and paternal health status	Healthy or having systemic/underlying disease
11	Maternal and paternal educational levels	≤Middle school, high school, or ≥college
12	Marital status of the parents	Consanguineous or nonconsanguineous
13	Degree of consanguinity	First-degree, second-degree, or distant relative
14	History of prenatal ultrasound utilization	Yes or no
15	Time of diagnosis	During pregnancy or after delivery
16	Type of delivery	Vaginal or cesarean section
17	Birth order	1^st^, 2^nd^, 3^rd^, and 4^th^ or higher
18	Birth weight	<2500 g, 2500-4000 g, or >4000 g
19	Maternal predisposing factors (during the first trimester of pregnancy)	Yes or no
20	Types of predisposing factors (during the first trimester of pregnancy)	Medications, stress, premature birth, history of abortion, history of multiple births, malnutrition, infectious disease, alcohol consumption, radiation exposure, or active smoking
21	Maternal passive smoking (during the first trimester of pregnancy)	Yes or no
22	Familial history of cleft lip and/or palate	Yes or no
23	Familial history of other congenital abnormalities	Yes or no

**Table 2:** Frequency of CL, CP, and CLP subtypes.

Cleft lip and/or palate classifications			
Cleft lip classification			
Cleft lip (*N* = 140, 100%)	Incomplete (*N* = 111, 79.3%)	Unilateral (*N* = 100, 71.4%)	Left (*N* = 58, 41.4%)
			Right (*N* = 37, 26.4%)
			Central (*N* = 5, 3.6%)
		Bilateral (*N* = 11, 7.9%)	—
	Complete (*N* = 29, 20.7%)	Unilateral (*N* = 23, 16.4%)	Left (*N* = 9, 6.4%)
			Right (*N* = 13, 9.3%)
			Central (*N* = 1, 0.7%)
		Bilateral (*N* = 6, 4.3%)	—

Cleft palate classification			
Cleft palate (*N* = 522, 100%)	Submucosal (*N* = 100, 19.3%)	—	—
	Incomplete (*N* = 416, 79.5%)	—	—
	Complete (*N* = 6, 1.2%)	Unilateral (*N* = 6, 1.2%)	Left (*N* = 4, 0.8%)
			Right (*N* = 2, 0.4%)
			Central (*N* = 0, 0%)
		Bilateral (*N* = 0, 0%)	

Cleft lip and palate classification			
Cleft lip and palate (*N* = 619, 100%)	—	Unilateral (*N* = 432, 69.8%)	Left (*N* = 271, 43.8%)
			Right (*N* = 154, 24.9%)
			Central (*N* = 7, 1.1%)
	—	Bilateral (*N* = 187, 30.2%)	—

**Table 3:** Gender distribution among subtypes of (A, B) CL, (C) CP, and (D) CLP.

	Gender				Total	
	Male		Female			
	*N*	%	*N*	%	*N*	%
(A) Cleft lip classification						
Incomplete cleft	62	44.3%	49	35%	111	79.3%
Complete cleft	16	11.4%	13	9.3%	29	20.7%
Total	78	55.7%	62	44.3%	140	100%

(B) Cleft lip classification						
Left unilateral cleft	40	28.6%	27	19.3%	67	47.9%
Right unilateral cleft	24	17.1%	26	18.6%	50	35.7%
Central unilateral cleft	2	1.4%	4	2.9%	6	4.3%
Bilateral cleft	12	8.6%	5	3.5%	17	12.1%
Total	78	55.7%	62	44.3%	140	100%

(C) Cleft palate classification						
Submucosal cleft	48	9.2%	52	10%	100	19.2%
Incomplete cleft	173	33.1%	243	46.5%	416	79.6%
Complete cleft	1	0.2%	5	1%	6	1.2%
Total	222	42.5%	300	57.5%	522	100%

(D) Cleft lip and palate classification						
Left unilateral cleft	168	27.1%	103	16.6%	271	43.7%
Right unilateral cleft	94	15.2%	60	9.7%	154	24.9%
Central unilateral cleft	5	0.8%	2	0.3%	7	1.1%
Bilateral cleft	113	18.3%	74	12%	187	30.3%
Total	380	61.4%	239	38.6%	619	100%

**Table 4:** Frequency of patients with CL, CP, and CLP in terms of their gender, birth dates, and birth season.

Variables	Cleft lip (*N* = 140)		Cleft palate (*N* = 522)		Cleft lip and palate (*N* = 619)		*P* value	Total (*N* = 1281)	
	*N*	%	*N*	%	*N*	%		*N*	%
Gender									
Male	78	55.7%	222	42.5%	380	61.4%	≤0.001^∗^	680	53.1%
Female	62	44.3%	300	57.5%	239	38.6%		601	46.9%
Birth dates									
≤1998	9	6.4%	45	8.6%	60	9.7%	≤0.001^∗^	114	8.9%
1999-2002	6	4.3%	26	5%	46	7.4%		78	6.1%
2003-2006	8	5.7%	27	5.2%	92	14.9%		127	9.9%
2007-2010	12	8.6%	60	11.5%	104	16.8%		176	13.7%
2011-2014	20	14.3%	133	25.5%	125	20.2%		278	21.7%
2015-2018	51	36.4%	147	28.2%	124	20%		322	25.2%
2019-2022	34	24.3%	84	16%	68	11%		186	14.5%
Birth season									
Spring	42	30%	124	23.8%	165	26.7%	0.779	331	25.8%
Summer	31	22.1%	141	27%	154	24.9%		326	25.4%
Fall	33	23.6%	129	24.7%	150	24.2%		312	24.4%
Winter	34	24.3%	128	24.5%	150	24.2%		312	24.4%

^∗^Significant results (*P* ≤ 0.05).

**Table 5:** Frequency of patients with CL/P in terms of their birthplace.

Birthplace (province)	Cleft lip and/or palate (*N* = 1281)	
	*N*	%
Fars	960	75%
Boushehr	117	9.1%
Kohgiluyeh and Boyer-Ahmad	82	6.4%
Hormozgan	74	5.8%
Khoozestan	48	3.7%

**Table 6:** Frequency of patients with CL, CP, and CLP in terms of their parental-related variables.

Variables	Cleft lip (*N* = 140)		Cleft palate (*N* = 522)		Cleft lip and palate (*N* = 619)		*P* value	Total (*N* = 1281)	
	*N*	%	*N*	%	*N*	%		*N*	%
Maternal age									
≤24	29	20.7%	125	23.9%	200	32.3%	0.013^∗^	354	27.6%
25-29	48	34.3%	183	35.1%	206	33.3%		437	34.1%
30-34	43	30.7%	133	25.5%	138	22.3%		314	24.5%
≥35	20	14.3%	81	15.5%	75	12.1%		176	13.7%
Paternal age									
≤24	9	6.4%	29	5.6%	55	8.9%	0.003^∗^	93	7.3%
25-29	22	15.7%	127	24.3%	164	26.5%		313	24.4%
30-34	61	43.6%	158	30.3%	190	30.7%		409	31.9%
≥35	48	34.3%	208	39.8%	210	33.9%		466	36.4%
Maternal health status									
Healthy	114	81.4%	418	80.1%	515	83.2%	0.395	1047	81.7%
Having systemic or underlying disease	26	18.6%	104	19.9%	104	16.8%		234	18.3%
Paternal health status									
Healthy	135	96.4%	479	91.8%	562	90.8%	0.900	1176	91.8%
Having systemic or underlying disease	5	3.6%	43	8.2%	57	9.2%		105	8.2%
Maternal educational level									
≤Middle school	50	35.7%	244	46.7%	315	50.9%	0.022^∗^	609	47.5%
High school	50	35.7%	155	29.7%	179	28.9%		384	30%
≥College	40	28.6%	123	23.6%	125	20.2%		288	22.5
Paternal educational level									
≤Middle school	54	38.6%	234	44.8%	299	48.3%	0.158	587	45.8%
High school	48	34.3%	163	31.2%	197	31.8%		408	31.9%
≥College	38	27.1%	125	23.9%	123	19.9%		286	22.3%
Marital status									
Consanguineous	73	52.1%	318	60.9%	364	58.8%	0.172	755	58.9%
Nonconsanguineous	67	47.9%	204	39.1%	255	41.2%		526	41.1%

^∗^Significant results (*P* ≤ 0.05).

**Table 7:** Frequency of patients with CL, CP, and CLP in terms of birth-related variables.

Variables	Cleft lip (*N* = 140)		Cleft palate (*N* = 522)		Cleft lip and palate (*N* = 619)		*P* value	Total (*N* = 1281)	
	*N*	%	*N*	%	*N*	%		*N*	%
History of prenatal ultrasound utilization									
Yes	109	77.9%	459	87.9%	521	84.2%	0.009^∗^	1089	85%
No	31	22.1%	63	12.1%	98	15.8%		192	15%
Time of diagnosis									
During pregnancy	25	17.9%	33	6.3%	105	17%	≤0.001^∗^	136	12.7%
After delivery	115	82.1%	489	93.7%	514	83%		1118	87.3%
Type of delivery									
Vaginal	67	47.9%	243	46.6%	344	55.6%	0.007^∗^	654	51.1%
Cesarean section	73	52.1%	279	53.4%	275	44.4%		627	48.9%
Birth order									
1^st^	53	37.9%	205	39.3%	252	40.7%	0.384	510	39.8%
2^nd^	51	36.4%	163	31.2%	212	34.2%		426	33.3%
3^rd^	24	17.1%	93	17.8%	83	13.4%		200	15.6%
4^th^ or higher	12	8.6%	61	11.7%	72	11.6%		145	11.3%
Birth weight									
<2500 g	14	10%	100	19.2%	99	16%	0.042^∗^	213	16.6%
2500-4000 g	116	82.9%	399	76.4%	477	77.1%		992	77.4%
>4000 g	10	7.1%	23	4.4%	43	6.9%		76	5.9%

^∗^Significant results (*P* ≤ 0.05).

**Table 8:** Frequency of patients with CL, CP, and CLP in terms of maternal- and familial-related variables.

Variables	Cleft lip (*N* = 140)		Cleft palate (*N* = 522)		Cleft lip and palate (*N* = 619)		*P* value	Total (*N* = 1281)	
	*N*	%	*N*	%	*N*	%		*N*	%
Maternal predisposing factors (during the first trimester of pregnancy)									
Yes	120	85.7%	457	87.5%	503	81.3%	0.013^∗^	1080	84.3%
No	20	14.3%	65	12.5%	116	18.7%		201	15.7%
Maternal passive smoking (during the first trimester of pregnancy)									
Yes	54	38.6%	176	33.7%	197	31.8%	0.302	427	33.3%
No	86	61.4%	346	66.3%	422	68.2%		854	66.7%
Familial history of CL/P									
Yes	43	30.7%	119	22.8%	180	29.1%	0.030^∗^	342	26.7%
No	97	69.3%	403	77.2%	439	70.9%		939	73.3%
Familial history of other congenital abnormalities									
Yes	3	2.1%	15	2.9%	18	2.9%	0.879	36	2.8%
No	137	97.9%	507	97.1%	601	97.1%		1245	97.2%

^∗^Significant results (*P* ≤ 0.05).

## Data Availability

The data used to support the findings of this study are available from the corresponding author upon reasonable request.

## Supplementary Materials

Supplementary Materials Supplementary Figure 1: the southern provinces of Iran and the regional distribution of their counties.
