# Supplementary material for: Epidemiological and Spatiotemporal Descriptive Analysis of Patients with Nonsyndromic Cleft Lip and/or Palate: A 12-Year Retrospective Study in Southern Iran
Source: Biomed Res Int. 2023 Apr 19;2023:7624875. doi: 10.1155/2023/7624875 (PMC10132907; doi:10.1155/2023/7624875)
Supplement: Supplementary Materials — Supplementary Figure 1: the southern provinces of Iran and the regional distribution of their counties. [file 7624875.f1.docx]

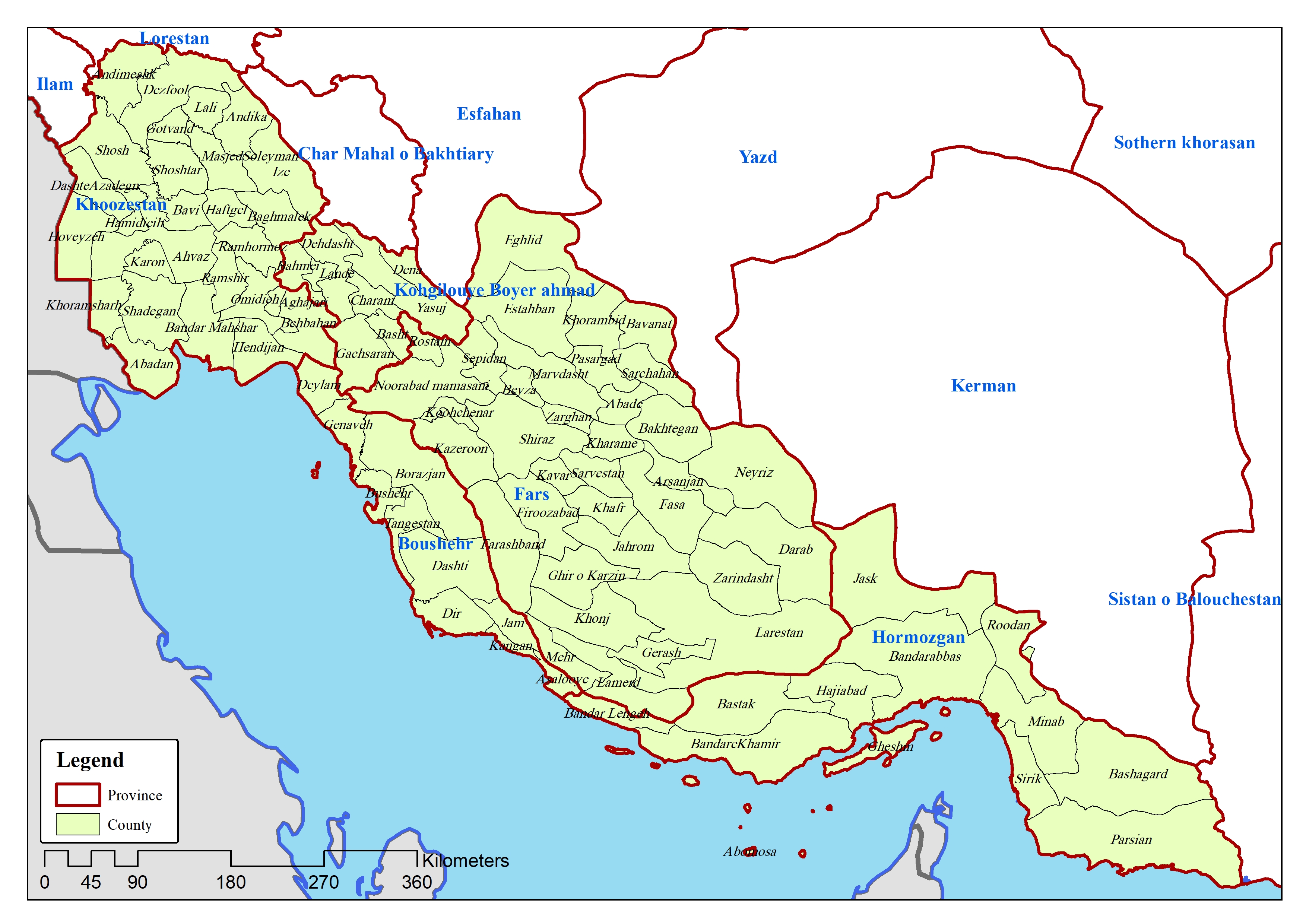


**Supplementary Figure 1:** The southern provinces of Iran and the regional distribution of their counties.
